# A Short Cognitive and Neuropsychiatric Assessment Scale for Progressive Supranuclear Palsy

**DOI:** 10.1002/mdc3.14348

**Published:** 2025-01-27

**Authors:** Sonja Porsche, Martin Klietz, Stephan Greten, Ines A. Piot, Ida Jensen, Florian Wegner, Lan Ye, Lea Krey, Matthias Höllerhage, Monika Pötter‐Nerger, Molly Zeitzschel, Keno Hagena, Jan Kassubek, Patrick Süß, Jürgen Winkler, Daniela Berg, Steffen Paschen, Lars Tönges, Doreen Gruber, Florin Gandor, Wolfgang H. Jost, Andrea A. Kühn, Inga Claus, Tobias Warnecke, David J. Pedrosa, Carsten Eggers, Claudia Trenkwalder, Joseph Classen, Johannes Schwarz, Alfons Schnitzler, Patricia Krause, Anja Schneider, Moritz Brandt, Björn Falkenburger, Inga Zerr, Mathias Bähr, Endy Weidinger, Johannes Levin, Sabrina Katzdobler, Emrah Düzel, Wenzel Glanz, Stefan Teipel, Ingo Kilimann, Johannes Prudlo, Thomas Gasser, Kathrin Brockmann, Annika Spottke, Anna Esser, Gabor C. Petzold, Gesine Respondek, Günter U. Höglinger

**Affiliations:** ^1^ Department of Neurology Hannover Medical School Hannover Germany; ^2^ German Center for Neurodegenerative Diseases (DZNE) Munich Germany; ^3^ Department of Neurology LMU University Hospital, LMU Munich Munich Germany; ^4^ Department of Neurology University Medical Center Hamburg‐Eppendorf Hamburg Germany; ^5^ Department of Neurology University of Ulm Ulm Germany; ^6^ German Center for Neurodegenerative Diseases (DZNE) Ulm Germany; ^7^ Department of Molecular Neurology University Hospital Erlangen, Friedrich‐Alexander‐Universität Erlangen‐Nürnberg Erlangen Germany; ^8^ Center of Rare Diseases Erlangen (ZSEER), University Hospital Erlangen, Friedrich‐Alexander‐Universität Erlangen‐Nürnberg Erlangen Germany; ^9^ Department of Neurology Kiel University Kiel Germany; ^10^ Department of Neurology St. Josef‐Hospital, Ruhr University Bochum Bochum Germany; ^11^ Neurodegeneration Research, Protein Research Unit Ruhr (PURE), Ruhr University Bochum Bochum Germany; ^12^ Movement Disorders Hospital, Beelitz‐Heilstätten Beelitz‐Heilstätten Germany; ^13^ Department of Neurology Otto‐von‐Guericke University Magdeburg Magdeburg Germany; ^14^ Parkinson‐Klinik Ortenau Wolfach Germany; ^15^ Movement Disorder and Neuromodulation Unit, Department of Neurology Charité, University Medicine Berlin Berlin Germany; ^16^ German Center for Neurodegenerative Diseases (DZNE) Berlin Germany; ^17^ Department of Neurology with Institute of Translational Neurology University Hospital Muenster Muenster Germany; ^18^ Department of Neurology and Neurorehabilitation Klinikum Osnabrueck Osnabrueck Germany; ^19^ Department of Neurology University Hospital of Marburg Marburg Germany; ^20^ Department of Neurology Knappschaftskrankenhaus Bottrop Bottrop Germany; ^21^ Paracelsus‐Elena Klinik Kassel Germany; ^22^ Department of Neurology Leipzig University Medical Center Leipzig Germany; ^23^ Department of Neurology Klinik Haag I. OB Mühldorf a. Inn Germany; ^24^ Institute of Clinical Neuroscience and Medical Psychology, and Department of Neurology Heinrich Heine University Düsseldorf Düsseldorf Germany; ^25^ German Center for Neurodegenerative Diseases (DZNE) Bonn Germany; ^26^ Department of Neurodegenerative Diseases and Geriatric Psychiatry University Hospital Bonn Bonn Germany; ^27^ German Center for Neurodegenerative Diseases (DZNE) Dresden Germany; ^28^ Department of Neurology University Hospital Carl Gustav Carus, Technische Universität Dresden Dresden Germany; ^29^ German Center for Neurodegenerative Diseases (DZNE) Goettingen Germany; ^30^ Department of Neurology University Medical Center, Georg August University Göttingen Germany; ^31^ Cluster of Excellence Nanoscale Microscopy and Molecular Physiology of the Brain (CNMPB), University Medical Center Göttingen Göttingen Germany; ^32^ Department of Neurology University Hospital of Munich, Ludwig‐Maximilians‐Universität (LMU) Munich Munich Germany; ^33^ Munich Cluster for Systems Neurology (SyNergy) Munich Munich Germany; ^34^ German Center for Neurodegenerative Diseases (DZNE) Magdeburg Germany; ^35^ Institute of Cognitive Neurology and Dementia Research, Otto‐von‐Guericke University Magdeburg Germany; ^36^ Institute of Cognitive Neuroscience, University College London London United Kingdom; ^37^ Clinic for Neurology, Medical Faculty, University Hospital Magdeburg Magdeburg Germany; ^38^ German Center for Neurodegenerative Diseases (DZNE) Rostock‐Greifswald Germany; ^39^ Department of Psychosomatic Medicine Rostock University Medical Center Rostock Germany; ^40^ Department of Neurology University Medical Centre Rostock Germany; ^41^ German Center for Neurodegenerative Diseases (DZNE) Tübingen Germany; ^42^ Hertie Institute for Clinical Brain Research, Department of Neurodegenerative Diseases University of Tübingen Tübingen Germany; ^43^ Center of Neurology, University Hospital Bonn Bonn Germany

**Keywords:** Progressive Supranuclear Palsy, 4‐repeat tauopathies, cognition, depression, quality of life

## Abstract

**Background:**

Patients with Progressive Supranuclear Palsy (PSP) suffer from several neuropsychological impairments. These mainly affect the frontal lobe and subcortical brain structures. However, a scale for the assessment of cognitive and neuropsychiatric disability in PSP is still missing.

**Objectives:**

To create and validate a new scale for cognitive and neuropsychiatric impairment in PSP.

**Methods:**

The Short Cognitive and Neuropsychiatric (ShoCo) scale was developed containing five items (bradyphrenia, apathy, aphasia, dysexecution and disinhibition). Each item can be categorized into 0 = no deficit, 1 = mild deficit, 2 = moderate deficit and 3 = severe deficit. The total score includes 15 points, 0 meaning no deficit and 15 severe deficits. Cross‐sectional and longitudinal data from 201 baseline and 71 follow up patients were analyzed.

**Results:**

Baseline ShoCo scale results were 5.9 ± 2.9. No significant differences between patients with Richardson syndrome (PSP‐RS) and variants (vPSP) could be detected in the PSP‐ShoCo scale scores (PSP‐RS 6.1 ± 3.0, n = 160, vPSP 5.1 ± 2.6, n = 41, *P* = 0.057). The scale showed good correlation with established scores (eg, Montreal cognitive assessment r = −0.535, *P* = 0.001). The ShoCo scale showed significant annualized change within the PSP‐RS patients (baseline 6.2 ± 2.9, follow up 6.9 ± 3.1, annualized diff. 1.0 ± 3.1, n = 57, *P* = 0.022).

**Conclusions:**

The ShoCo scale seems a promising and valid tool to measure specific neuropsychological disabilities of PSP patients in clinical routine and research.

Progressive Supranuclear Palsy (PSP) is an atypical Parkinsonian syndrome with supranuclear gaze palsy, early postural instability and cognitive impairments.^1^, ^2^ The Movement Disorder Society diagnosis criteria for PSP established a large phenotypic spectrum of patients with high likelihood of PSP specific 4‐repeat tauopathy.^3^ PSP patients are confronted with reduced life expectancy and loss of autonomy early in the course of the disease.^4^, ^5^ Patients with PSP suffer from a large variety of cognitive and neuropsychiatric impairments predominantly reflecting frontal lobe dysfunction, but also temporal lobe and subcortical features contribute to cognitive decline.^6^, ^7^ Bradyphrenia, dysexecution, disinhibition, apathy and aphasia are the disease specific cognitive and neuropsychiatric components in patients with PSP^6^ dramatically reducing their quality of life.^8^, ^9^, ^10^ Several scales and questionnaires like Montreal cognitive assessment (MoCA), the Starkstein Apathy Scale (SAS), PSP Rating Scale (PSPRS), PSP quality of life scale (PSP QoL) can be used to detect and quantify these symptoms of the disease.^11^, ^12^ Nevertheless, usage of several different scales is time consuming and may overstrain most PSP patients. Therefore, a scale for PSP specific cognitive and neuropsychiatric impairments is still an unmet need.

Aim of this study was to establish a short and useful scale for clinical routine applications and trials with regard to cognitive and neuropsychiatric symptoms in PSP. This Short Cognitive and Neuropsychiatric (ShoCo) scale for PSP patients has been created and validated in two multicenter prospective longitudinal PSP cohorts from Germany.

## Methods

### Scale Development

The ShoCo scale was developed by analyzing the cognitive and neuropsychiatric deficits of PSP patients. To identify the main cognitive and neuropsychiatric symptoms of PSP patients, a systematic literature review was conducted. Publications from 1992 until 2020 from PubMed, Medline and Cochrane were included. Based on this review, the following five symptoms mainly contributed to cognitive and neuropsychiatric dysfunction in PSP: bradyphrenia, apathy, aphasia, dysexecution and disinhibition. By identifying these symptoms in already established scores for PSP patients and comparing alternative items for each symptom (see Table S1), the following items were combined: PSPRS item 9 represents bradyphrenia, its interference with the Activities of Daily Living (ADL) is scored from 0 to 4. A higher number indicates a higher interference with ADL.^13^ This item was chosen based on a good sensitivity to change.^12^ The SAS score item 1 represents apathy, where the caregiver or a relative has to rate how much interest the patient has in learning new things. A score of 3 means no interest at all, 0 means high interest.^14^ The Frontal Assessment Battery (FAB) subtest lexical fluency represents aphasia, where the patient has to name as many words starting with the letter “s” in 60 seconds as he/she can.^15^ The Luria sequence as another subtest of the FAB represents dysexecutive symptoms. The examiner shows the patient a specific sequence with both hands (fist, cut, slap) three times before the patient has to imitate the examiner. The patients score 3 if he/she is able to repeat the sequence correctly for more than 6 times. A lower score represents lower dysexecutive function. To test for disinhibition, the three‐clap test was used. The patient has to clap for exactly three times (score = 3). A higher number of claps corresponds to a lower score (>10 claps equal score = 0).^15^

The original values from these scores were converted into the ShoCo scale. Each item can be categorized into 0 = no deficit, 1 = mild deficit, 2 = moderate deficit and 3 = severe deficit. The total score includes 15 points, 0 meaning no deficit and 15 severe deficit (see Table 4).

### Participants and Assessment

Ethical approvals were obtained from the local Ethics Committees of all participating study centers. The data analysis of the study was additionally amended to the Ethics Committee at Hannover Medical School (No. 3558‐2017, amendment in 2020).

Longitudinal and cross‐sectional data from two German multicentric cohort studies were analyzed; The DescribePSP network from the German Center for Neurodegenerative Diseases and the ProPSP network.^16^ The main inclusion criterion was the presence of complete data to form the ShoCo scale.

In total 357 baseline and 148 follow up patients were evaluated. After excluding patients with incomplete data for the ShoCo scale, 201 baseline patients (116 ProPSP, 85 DescribePSP) and 71 follow ups (49 ProPSP, 22 DescribePSP) were included in the ShoCo scale analyses. The following section illustrates the phenotypic distribution:

Probable PSP: PSP‐RS 159 (79.1%), PSP‐P 21 (10.4%), PSP‐F 4 (2%); Possible PSP: PSP‐OM 2 (1%), PSP‐PGF 1 (0.5%), PSP SL 1 (0.5%); Suggestive of PSP: PSP‐P 3 (1.5%), PSP‐PI 2 (1%), PSP‐RS 1 (0.5%), PSP‐CBS 1 (0.5%), PSP‐OM 1 (0.5%).

To check for sensitivity to change the baseline and follow up data from 57 PSP‐RS patients were included. The classification into phenotypes (PSP‐RS and vPSP) was performed according to the MDS criteria.^3^

In order to obtain a comparison of the ShoCo scale with already established PSP scores, data of the Schwab and England Activities of Daily Living (SEADL), Geriatric Depression Scale (GDS‐15), SAS, Clinical Global Impression Scale of Severity (CGI‐S), MoCA, PSP Staging System (PSPSS), PSPRS, modified PSP Rating Scale (mPSPRS) and PSP Clinical Deficit Scale (PSP‐CDS) were included in the analysis.

The PSPRS scale is a 28‐item scale to evaluate the presence and progression of PSP symptoms. The scale is divided into six categories: history, mental (including the item bradyphrenia), bulbar, ocular motor exam, limb and gait/midline exam. The scale ranges from 0 to 100, a higher number meaning higher deficit.^13^ The PSPSS evaluates gait and the ability to walk in a five‐point scale: gait and stability normal (scale 1) to no useful gait, but patient may be able to remain standing unassisted or transfer between chair and bed (scale 5). In addition to the PSPRS we included the mPSPRS. After previous reevaluation the 28 items from the PSPRS, seven items (irritability, sleep difficulty, grasping/imitative/utilizing behavior, voluntary left and right saccades, finger tapping, toe tapping and postural kinetic or rest tremor) were excluded from the mPSPRS. The mPSPRS can be used for measuring the progression of PSP patients.^11^

To evaluate the independence of daily living we used the SEADL scale. Complete independence equals 100% in the scale. The lower the percentage the less independent the patient is.

GDS‐15 was used to assess the depressive symptoms of the patients.^17^, ^18^ Values of 6 or more are suggestive for depression.

Further, apathy in patients was assessed by their caregivers using the 14‐item SAS.^14^

For the overall severity of illness and impairment we used CGI‐S ranging from 1 “normal, not at all ill” to 7 “among the most extremely ill patients”.

The MoCA score was used as a cognitive screening test, ranging from 0 to 30 points (30 to 26 points were considered as normal cognitive function, 25 to 21 points as mild cognitive impairment and below 21 points as suspicious for dementia).^19^, ^20^

The PSP QoL scale evaluates the physical (22 items) and mental (23 items) impact of PSP on the quality of life. The scale ranges from 0 to 180 points, higher points referring to lower quality of life.^21^

Lastly, we included the PSP CDS scale. The 7 items (Akinesia‐rigidity, Bradyphrenia, Communication, Dysphagia, Eye movements, Finger dexterity, and Gait & balance) range from 0 = no deficit to 3 = severe deficit.^12^

### Statistical Analysis

All statistical analyses were performed using IBM SPSS Statistics Version 29 for Macintosh (IBM Corp., New York, NY, USA). Results are presented as mean, standard deviation (SD) and range.

Shapiro–Wilk test and Kolmogorov–Smirnov‐test were used to test for normal distribution. The Mann–Whitney *U* test was applied to detect significant differences between the phenotypes within the baseline population, since these variables were not normally distributed. An outlier analysis was performed, however, extreme values without cause of error were not excluded. Significance within the annualized data was tested with the Wilcoxon sign rank test. *P* < 0.05 was set as a level of significance. The annualized difference was used for power calculations (80% power, two‐sample *t* test and Mann Whitney *U* test in parenthesis) to measure standard effect size (mean divided by SD) and estimated sample size needed for 30% and 50% change. Nonparametric Spearman correlation was applied to detect correlation between the ShoCo scale and established scores. Bonferroni correction was applied here to correct for multiple testing.

Internal consistency was measured by Cronbach's alpha and item‐total scale correlations.

## Results

### Baseline and Phenotype Differences

The descriptive statistics of the 201 baseline patients is presented in Table 1. The total ShoCo scale resulted in a mean of 5.9 ± 2.9 (n = 201, min = 0, max = 13). The highest deficit could be seen in the item aphasia (n = 201; 1.5 ± 1.0; min = 0, max = 3) and the mildest deficit was the item disinhibition (n = 201, 0.6 ± 0.9, min = 0, max = 3). Patients with PSP‐RS showed slightly but not significantly more deficits in the ShoCo scale (n = 160, 6.1 ± 3.0) then vPSP (n = 41, 5.1 ± 2.6; *P* = 0.057). Significant differences between PSP‐RS and vPSP were detected within the SEADL (*P* = 0.009), PSPSS (*P* = 0.001), PSPRS (*P* = 0.001), mPSPRS (*P* = 0.001) and PSP‐CDS (*P* = 0.009), as well as for the items apathy (*P* = 0.046), aphasia (*P* = 0.031) and disinhibition (*P* = 0.018).

**TABLE 1:** Demographic and clinical data of the study population at baseline

PSP phenotypes baseline		N	All	PSP‐RS	vPSP
			n = 201	n = 160	n = 41
Gender m/f (%)		201	106/95 (52.7%/47.3%)	79/81 (49.4%/50.6%)	27/14 (65.9%/34.1%)
Age at examination (y)		201	69.8 ± 7.2 (51–89)	70.1 ± 7.2 (51–89)	68.4 ± 7.2 (53–83)
Disease duration (y)		199	4.0 ± 2.8 (0–17.7)	4.2 ± 2.9 (0–17.6)	3.6 ± 2.5 (0–10.7)
SEADL		200	60.7 ± 21.5 (10–100)	58.7 ± 20.6 (10–90)**	68.3 ± 23.4 (20–100)**
GDS		194	6.4 ± 4.1 (0–15)	6.4 ± 4.0 (0–15)	6.5 ± 4.3 (0–15)
SAS		192	18.3 ± 7.7 (0–41)	18.2 ± 7.8 (0–41)	18.7 ± 7.4 (3–35)
CGI‐S		199	4.2 ± 1.1 (0–9)	4.3 ± 1.1 (0–9)	4.1 ± 1.2 (2–6)
MoCA		192	21.5 ± 5.2 (3–29)	21.4 ± 5.2 (3–29)	21.8 ± 5.3 (10–28)
PSP‐QoL		179	38.1 ± 17.8 (6.1–95.6)	38.9 ± 17.8 (6.1–95.6)	35.2 ± 17.5 (6.3–64.8)
PSPSS		197	3.0 ± 1.0 (1–5)	3.1 ± 1.0 (1–5)**	2.5 ± 0.9 (1–4)**
PSPRS		198	33.6 ± 12.8 (0–68)	35.1 ± 12.5 (0–68)**	27.6 ± 12.4 (9–58)**
mPSPRS		201	7.4 ± 5.1 (0–20)	8.2 ± 5.0 (0–20)**	4.4 ± 4.3 (0–16)**
PSP‐CDS		113	7.3 ± 3.1 (0–16)	7.8 ± 2.9 (0–16)**	6.1 ± 3.2 (0–15)**
PSP‐ShoCo	Total	201	5.9 ± 2.9 (0–13)	6.1 ± 3.0 (0–13)	5.1 ± 2.6 (1–11)
	Bradyphrenia	201	1.2 ± 0.9 (0–3)	1.2 ± 0.8 (0–3)	1.0 ± 0.9 (0–3)
	Apathy	201	1.3 ± 1.0 (0–3)	1.2 ± 1.0 (0–3)*	1.5 ± 0.8 (0–3)*
	Aphasia	201	1.5 ± 1.0 (0–3)	1.6 ± 1.1 (0–3)*	1.2 ± 0.9 (0–3)*
	Dysexecution	201	1.4 ± 1.1 (0–3)	1.4 ± 1.1 (0–3)	1.1 ± 1.1 (0–3)
	Disinhibition	201	0.6 ± 0.9 (0–3)	0.7 ± 0.9 (0–3)*	0.3 ± 0.7 (0–3)*

Data are given as mean ± standard deviation (range). Mann Whitney *U* test was applied to test for significant difference between Richardson's syndrome and vPSP.

Abbreviations: y, years; m/f, male/female; n, number of patients; PSP‐RS, Progressive Supranuclear Palsy with predominant Richardson's syndrome; vPSP, variant PSP phenotypes; SEADL, Schwab and England Activities of Daily Living Scale; GDS, Geriatric Depression Scale; SAS, Starkstein Apathy Scale; CGI‐S, Clinical Global Impression‐Severity scale; MoCA, Montreal Cognitive Assessment; PSP‐QoL, Progressive Supranuclear Palsy Quality of Life scale; PSPSS, PSP Staging System; PSPRS, Progressive Supranuclear Palsy Rating Scale; PSP‐CDS, Progressive Supranuclear Palsy Clinical Deficits Scale; mPSPRS, modified Progressive Supranuclear Palsy Rating Scale; PSP‐ShoCo, Progressive Supranuclear Palsy Short Cognitive and neuropsychiatric Scale.

* *P* < 0.05.

** *P* < 0.01.

### Longitudinal Change

**Figure 1:**
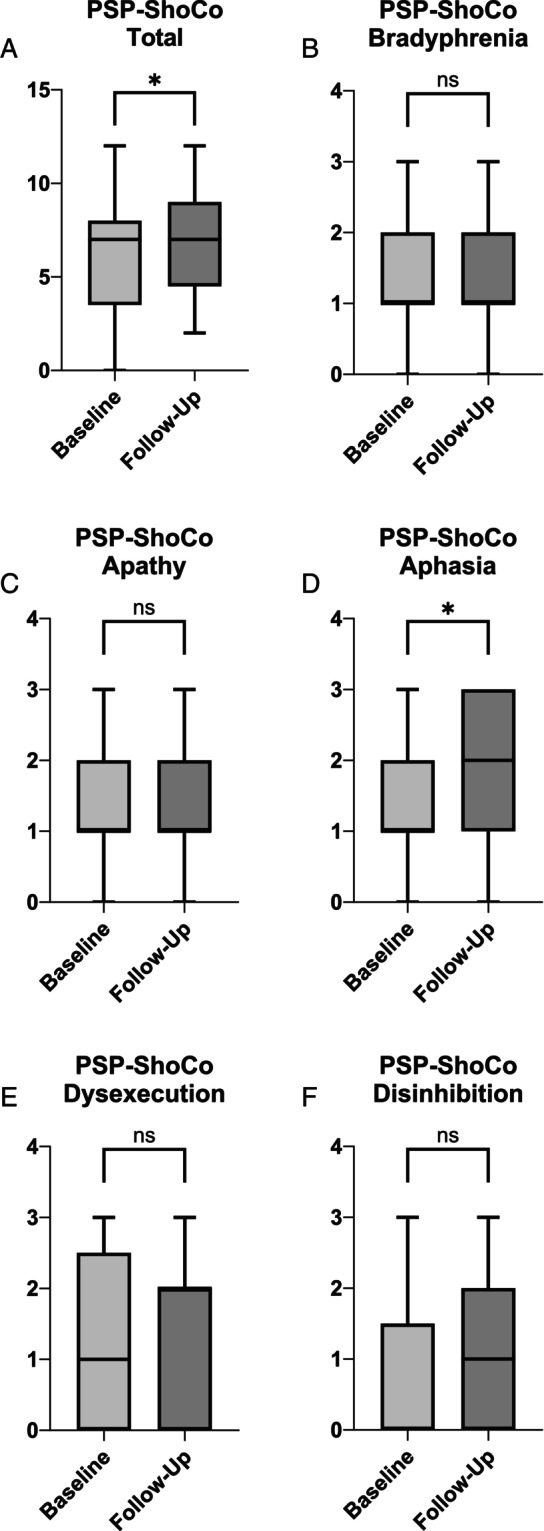
Displayed are box plots of the baseline and follow‐up data of the respective items (**B–F**) of the ShoCo scale and the total score (**A**). The whiskers span from minimum to maximum. The box spans from lower to upper quartile and beyond, presenting the median. *P* values are calculated with Wilcoxon sign rank test. *P < 0.05. ShoCo, Short Cognitive and Neuropsychiatric; ns, non significant.

Table 2 displays the baseline and follow up data of 57 PSP‐RS patients with annualized differences, *P* value and standardized effect size. Since the Richardson syndrome is the most common phenotype of PSP, we have focused on this patient group in the annualization. A significant annualized change was shown for the ShoCo scale (Fig. 1) in total (ann. diff. 1.0 ± 3.1; *P* = 0.022) as well as for the item aphasia (ann. diff. 0.4 ± 1.3; *P* = 0.021). The cognitive function of the patients did not change significantly within the other ShoCo scale items. Compared to other analyzed cognitive scales (eg, SAS or MoCA) the ShoCo scale revealed a much higher sensitivity to change (30% change: ShoCo n = 1546; SAS n = 8037; MoCA n = >10.000; 50% change: ShoCo n = 558; SAS n = 2894; MoCA n = 6932).

**TABLE 2:** Comparison between baseline and follow‐up: annualized change of PSP‐RS patients

Item		N	Values at		Annualized difference from baseline value		Standardized effect size	Sample size	Sample size
			Baseline	Follow‐up	Mean ± SD (min–max)	*P* value		30% change	50% change
Gender m/f (%)		57	34/23 (59.6%/40.4%)	34/23 (59.6%/40.4%)	n.a.	n.a.	n.a.	n.a.	n.a.
Age at examination (y)		57	69.2 ± 6.4 (56–85)	70.0 ± 6.4 (57–85)	n.a.	n.a.	n.a.	n.a.	n.a.
Disease duration at examination (y)		57	4.0 ± 2.6 (0–11.2)	4.9 ± 2.6 (0.70–11.8)	n.a.	n.a.	n.a.	n.a.	n.a.
SEADL		57	64.0 ± 19.6 (30–90)	54.9 ± 21.7 (20–90)	−12.7 ± 25.5 (−93.1 to 54.2)	**<0.001**	−0.497	709 (802)	256 (296)
GDS		56	5.6 ± 3.9 (0–15)	6.0 ± 3.7 (0–15)	0.9 ± 6.1 (−19.3 to 13.0)	0.102	0.153	7464 (8639)	2688 (3111)
SAS		54	18.0 ± 7.4 (3–41)	18.5 ± 8.4 (3–35)	1.4 ± 9.2 (−15.9 to 24.6)	0.320	0.147	8037 (9301)	2894 (3350)
CGI		57	4.1 ± 1.1 (0–6)	4.4 ± 1.1 (2–6)	0.5 ± 1.8 (−5.4 to 8.2)	0.054	0.268	2434 (2817)	877 (1015)
MoCA		56	22.3 ± 4.6 (9–28)	22.5 ± 5.1 (11–30)	−0.5 ± 5.2 (−13.0 to 11.5)	0.843	−0.095	>10,000 (>10,000)	6932 (8023)
PSP‐QoL		53	36.2 ± 18.1 (6.1–95.6)	37.9 ± 16.4 (10.2–75.0)	4.6 ± 29.6 (−66.5 to 76.1)	0.153	0.154	7315 (8467)	2634 (3049)
PSPSS		55	2.8 ± 1.0 (1–5)	3.3 ± 0.9 (1–5)	0.8 ± 1.4 (−1.9 to 5.8)	**<0.001**	0.571	536 (620)	194 (224)
PSPRS		57	32.5 ± 11.2 (12–56)	38.4 ± 12.8 (15–65)	9.2 ± 11.9 (−21.2 to 47.4)	**<0.001**	0.767	298 (345)	108 (125)
mPSPRS		57	7.1 ± 4.0 (1–16)	9.3 ± 5.2 (0–23)	3.34 ± 5.41 (−13.7 to 24.7)	**<0.001**	0.605	478 (553)	173 (200)
PSPCDS		38	6.6 ± 2.4 (0–13)	8.0 ± 3.2 (0–14)	2.4 ± 5.9 (−18.6 to 13.4)	**0.004**	0.416	1009 (1167)	364 (421)
PSP‐ShoCo	Total	57	6.2 ± 2.9 (0–12)	6.9 ± 3.1 (2–12)	1.0 ± 3.1 (−5.6 to 11.5)	**0.022**	0.336	1546 (1789)	558 (645)
	Bradyphrenia	57	1.3 ± 0.9 (0–3)	1.4 ± 0.7 (0–3)	0.2 ± 1.3 (−4.2 to 2.2)	0.186	0.140	8934 (>10,000)	3217 (3724)
	Apathy	57	1.4 ± 1.0 (0–3)	1.5 ± 1.0 (0–3)	0.3 ± 1.5 (−3.9 to 5.8)	0.236	0.188	4937 (5714)	1778 (2058)
	Aphasia	57	1.5 ± 1.1 (0–3)	1.7 ± 1.1 (0–3)	0.4 ± 1.3 (−3.3 to 3.8)	**0.021**	0.315	1754 (2031)	633 (732)
	Dysexecution	57	1.4 ± 1.1 (0–3)	1.4 ± 1.1 (0–3)	−0.1 ± 1.9 (−5.3 to 5.8)	0.527	−0.04	>10,000 (>10,000)	>10,000 (>10,000)
	Disinhibition	57	0.7 ± 0.9 (0–3)	0.9 ± 1.0 (0–3)	0.3 ± 1.2 (−3.9 to 2.8)	0.091	0.261	2557 (2960)	921 (1066)

Sample sizes required for a 2‐arm, 1‐year follow‐up therapeutic trial to detect 30% or 50% change. Data statistics at baseline and follow‐up from both PSP‐RS and vPSP combined as well as annualized rate of change, defined as follow‐up scale score minus baseline score divided by time in years, and power calculations. Estimated sample sizes needed to detect a 30% and 50% rate of change based on 80% power, 2‐sided, 2‐sample *t* test, were calculated. Approximations of the sample size for the Mann–Whitney *U* test are given in parentheses. *P* values are calculated with Wilcoxon sign rank test. Data are given as mean ± SD (range), unless indicated otherwise. A *p* value <0.05 was considered significant.

Abbreviations: y, years; m/f, male/female; n, number of patients; n.a., not applicable; SEADL, Schwab and England Activities of Daily Living Scale; GDS, Geriatric Depression Scale; SAS, Starkstein Apathy Scale; CGI‐S, Clinical Global Impression‐Severity scale; MoCA, Montreal Cognitive Assessment; PSP‐QoL, Progressive Supranuclear Palsy Quality of Life scale; PSPSS, PSP Staging System; PSP‐RS, Progressive Supranuclear Palsy Rating Scale; PSP‐CDS, Progressive Supranuclear Palsy Clinical Deficits Scale; mPSPRS, modified Progressive Supranuclear Palsy Rating Scale; PSP‐ShoCo, Progressive Supranuclear Palsy Short Cognitive and Neuropsychiatric Scale.

### Correlation and Internal Consistency

Baseline data were used to evaluate the correlation between the ShoCo scale and existing scales (Table 3). The SEADL score (all: r = −0.508, *P* = <0.001; PSP‐RS: r = −0.467, *P* < 0.001; vPSP: r = −0.577, *P* < 0.001) and MoCA (all: r = −0.535, *P* < 0.001; RS: r = −0.517, *P* < 0.001; vPSP: r = −0.644, *P* < 0.001) showed the highest correlation with the ShoCo scale throughout all phenotypes. vPSP showed a higher correlation than the PSP‐RS, except for mPSPRS, SAS and PSPSS. A robust difference between PSP‐RS and vPSP was seen in GDS‐15 (RS: r = 0.273, *P* < 0.001; vPSP: r = 0.531, *P* < 0.001). PSPRS showed strong correlation with the ShoCo scale (all: r = 0.451, *P* < 0.001; PSP‐RS: r = 0, 424, *P* < 0.001; vPSP: r = 0.492, *P* < 0.001), while the mPSPRS revealed a lower correlation (all: r = 0.342, *P* < 0.001; PSP‐RS: r = 0.332, *P* < 0.001; vPSP: r = 0.221, *P* > 0.005).

**TABLE 3:** Correlation between the ShoCo scale and other established scales for PSP at baseline

Baseline	SEADL	GDS	SAS	CGI‐S	MoCA	PSP‐QoL	PSPSS	PSPRS	mPSPRS	PSP‐CDS
All phenotypes	−0.508** n = 200	0.323** n = 194	0.379** n = 192	0.312** n = 199	−0.535** n = 192	0.306** n = 179	0.288** n = 197	0.451** n = 198	0.342** n = 201	0.409** n = 113
PSP‐RS	−0.467** n = 159	0.273** n = 154	0.395** n = 152	0.274** n = 159	−0.517** n = 154	0.258* n = 141	0.319** n = 157	0.424** n = 159	0.332** n = 160	0.395** n = 82
vPSP	−0.577** n = 41	0.531* n = 40	0.383 n = 40	0.448* n = 40	−0.644** n = 36	0.450 n = 38	0.004 n = 40	0.492* n = 39	0.221 n = 41	0.405 n = 31

Spearman's r correlation coefficients. N is the number of analyzed pairs. Bonferroni correction was applied here to correct for multiple testing. All correlations were statistically significant.

Abbreviations: y, years; m/f, male/female; n, number of patients; PSP‐RS, Progressive Supranuclear Palsy with predominant Richardson's syndrome; vPSP, variant PSP phenotypes; SEADL, Schwab and England Activities of Daily Living Scale; GDS, Geriatric Depression Scale; SAS, Starkstein Apathy Scale; CGI‐S, Clinical Global Impression‐Severity scale; MoCA, Montreal Cognitive Assessment; PSP‐QoL, Progressive Supranuclear Palsy Quality of Life scale; PSPSS, PSP Staging System; PSPRS, Progressive Supranuclear Palsy Rating Scale; PSP‐CDS, Progressive Supranuclear Palsy Clinical Deficits Scale; mPSPRS, modified Progressive Supranuclear Palsy Rating Scale; PSP‐ShoCo, Progressive Supranuclear Palsy Short Cognitive and Neuropsychiatric Scale.

* <0.005.

** <0.001.

**TABLE 4:** The PSP‐Short Cognitive and Neuropsychiatric (ShoCo) scale

ShoCo‐scale	Derivation	0 = No deficit	1 = Mild deficit	2 = Moderate deficit	3 = Severe deficit	Score
Bradyphrenia [BRA]	ShoCo item	No bradyphrenia	Equivocal or mild, but not interfering with Activities of Daily Living (ADL)	Interfering moderately with ADL	Interfering markedly with severely of daily living	
	PSPRS item 9	0 Clearly absent in ADL	1 Equivocal/minimal, not interfering with ADL 2 Clearly present, but not interfering with ADL	3 Interfering mildly with ADL	4 Interfering markedly with ADL	
Apathy [APA] (Is your relative interested in learning something new?	ShoCo item	0 A lot	1 Some	2 Slightly	3 Not at all	
	SAS item 1	0 A lot	1 Some	2 Slightly	3 Not at all	
Aphasia [APH] (letter S, 60 s)	ShoCo item	>9 Words	6–9 Words	3–5 Words	<3 Words	
	FAB lexical fluency	3 More than nine words	2 Six to nine words	1 Three to five words	0 Under three words	
Dysexecution [DYS] (Luria sequence)	ShoCo item	6 Correct series in a row	At least 3 correct series in a row	Fails by himself, 3 correct series in a row when aided	<3 Correct series in a row even when aided	
	FAB motor series	3 Correct series in a row	2 At least 3 correct series in a row	1 Fails by himself, 3 correct series in a row when aided	0 < 3 Correct series in a row even when aided	
Disinhibition [DIS] (Applaus sign)	ShoCo item	Patient claps 3 times	Patient claps 4 times	Patient claps 5–10 times	Patient claps >10 times	
	Three‐clap test	3 Patient claps 3 times	2 Patient claps 4 times	1 Patient claps 5–10 times	0 Patient claps >10 times	
					Total score	15

Values from the 5 items from the original scores and the converted values into the PSP‐ShoCo Scale.

Abbreviations: PSPRS, Progressive Supranuclear Palsy Rating Scale; SAS, Starkstein Apathy Scale; FAB, Frontal Assessment Battery; PSP‐ShoCo, Progressive Supranuclear Palsy Short Cognitive and Neuropsychiatric Scale.

The internal consistency with a Cronbach's alpha of 0.56 was moderate to low, which could be explained by the small number of items within the scale, each referring to a different symptom, but still acceptable. Excluding the item apathy would increase it to 0.6. Item‐total correlation confirmed that the item apathy is less consistent with the total score than other items, but still all items have significant item‐total correlation with *P* < 0.001 (n = 201, bradyphrenia: r = 0.574; apathy: r = 0.436; aphasia: r = 0.702; dysexecutive: r = 0.690; disinhibition: r = 0.567).

## Discussion

There is an urgent need for a disease specific scale for timely measure of neuropsychologic impairments in PSP. To establish a concise and user‐friendly cognitive and neuropsychiatric assessment for patients with PSP, the ShoCo scale was developed and the psychometric properties were validated. The primary focus during the scale development was to assess most relevant cognitive and neuropsychiatric impairments in PSP patients in a time efficient, precise and reliable fashion. The scale should be less burdensome for the PSP patients and easily applicable.

The main objectives of the study were to prove the capability of the ShoCo scale to detect cognitive and neuropsychiatric changes over time and to explore whether those changes correlate with other important outcome variables of PSP, such as the PSPRS. The ShoCo integrates five cognitive and neuropsychiatric symptom‐related items, each derived from a different existing scale, tailored specifically for PSP. The results of this study demonstrate moderate to strong correlations of the ShoCo scale with other scales relevant for the characterization of PSP like the PSPRS, MoCA, PSP‐CDS, GDS‐15 and SEADL.

People with PSP present with specific neuropsychological complaints impairing their quality of life. These deficits mainly affect the domains dysexecution, disinhibition, bradyphrenia, apathy and aphasia. A composite score for the measurement of these impairments has not been available yet. Individual tests were able to assess one or several aspects of neuropsychological impairment, but until now no test or scale was able to check all specific deficits together as a bedside examination.

In the PSPRS, a wide array of PSP‐specific symptoms, including mental deficits like bradyphrenia and apathy, are examined. However, key symptoms such as aphasia, disinhibition, and dysexecution are notably absent.^13^ Similarly, while modifications to the PSPRS have been made to align with patient‐relevant milestones of progressive impairment, cognitive items have not been incorporated into the process.^11^ Both scales allow a structured assessment of PSP symptoms and demonstrate a constant progression of symptoms over time.

Among existing rating scales, the FAB emerges as the most specific test for cognitive impairments in PSP, as it assesses dysexecution, disinhibition, and aphasia.^15^ However, studies suggest that utilizing subscores rather than the total FAB score might be more beneficial for detecting cognitive decline in PSP patients.^22^, ^23^, ^24^ For instance, Sitek and colleagues,^22^ studied 20 patients with PSP‐RS and found that the most common deficits within the FAB were in the motor series (dysexecution in the ShoCo scale) with 95% and in verbal fluency (aphasia in the ShoCo scale) with 80%. While these findings align with the pronounced cognitive deficit observed in PSP patients, our study revealed a higher prevalence of aphasia compared to dysexecutive symptoms. Moreover, longitudinal changes within the FAB have been detected in patients with frontotemporal lobar degeneration but not specifically in PSP patients.^25^

Stamelou and colleagues,^23^ investigated the utility of the FAB score in discriminating between PSP and Frontotemporal dementia (FTD), including 70 PSP, 103 FTD, 26 Parkinson's disease (PD) and 11 multiple system atrophy (MSA) patients. While no significant differences were observed between PSP and FTD, distinctions could be found between PSP, PD, and MSA. However, for longitudinal assessment the study only relied on the correlation between the FAB score and the disease duration and did not include longitudinal data. Further they found that the combination of the two subscores “verbal fluency” and “Luria sequence” was more useful for discrimination than the whole score itself, as both items were also scored significantly lower in PSP than in PD or MSA, aligning with a previous study.^26^ In agreement with that, Gerstenecker and colleagues,^27^ highlighted verbal fluency as the most indicative parameter of cognitive deficits within the FAB score among PSP patients. Nevertheless, it has not been investigated yet whether the two subscores can depict longitudinal progression of PSP better.^23^, ^26^ The results of this study align with Gerstenecker's statement, as verbal fluency has the highest scores within our scale.^27^

Aphasia shows a significant longitudinal change over time, which emphasizes its value as an indicator for the progression of PSP.^28^, ^29^ Despite this agreement with Stamelou and colleagues, we cannot confirm that dysexecution is a good indicator of longitudinal progression (*P* = 0.527). By looking at the baseline data and the different phenotypes, we confirmed a previous study, which indicated that dysexecution is more severe in PSP‐RS than in vPSP.^30^

Besides the FAB, the MoCA score is a common score for the assessment of cognitive impairment of PSP patients. However, the MoCA score does not include dysexecution and disinhibition. Cohort studies indicate that the MoCA lacks sensitivity for detecting longitudinal changes in cognition of PSP patients, which limits its utility for future interventional studies aiming at stabilization or improvement of cognition in PSP.^31^ For example, Pereira and colleagues,^32^ assessed longitudinal cognitive and early motor symptoms in 28 PSP patients and 28 healthy controls using the MoCA and MMSE. Confirming other studies^33^, ^34^, ^35^ the authors found that the MoCA score can be used to detect cognitive deficits in PSP baseline patients and is superior to the MMSE. However, the results showed a lack of the sensitivity to change within the MoCA score, corresponding to the findings of the CDS score^12^ and a lack of specificity for PSP patients.^36^ Similarly, our study failed to detect significant sensitivity to change within the MoCA score.

Pereira and colleagues,^32^ emphasized the need for a more suitable assessment of the longitudinal cognitive change of PSP patients. The ShoCo scale includes both dysexecution and disinhibition. Further, it shows a significant sensitivity to change (*P* = 0.022). Compared to the MoCA and SAS, the sample size for 30% and 50% change was much lower in the ShoCo scale.

Looking at the item disinhibition it is known to be more prevalent in PSP patients than in other conditions with frontotemporal degeneration.^37^ Some studies even found a 90% prevalence of disinhibition in PSP patients.^38^ Therefore, it is very important to include this symptom in the cognitive and neuropsychiatric assessment of PSP patients. We calculated a significant difference within the phenotypes, revealing worse disinhibition in PSP‐RS versus vPSP. In contrast to disinhibition, patients with PSP‐RS showed significantly less apathy than patients with vPSP and significantly higher aphasia, supporting a previous study.^39^

In the case of apathy, there are no uniform criteria for eliciting this symptom. However, it is known that PSP patients suffer from stronger apathy than patients with other atypical Parkinsonian syndromes.^7^ The internal consistency of the ShoCo scale would be enhanced from 0.56 to 0.6 by the exclusion of apathy. However, we believe that it is crucial to include apathy since it is a significant symptom of PSP and can be particularly useful in differentiating it from other frontotemporal diseases.^7^ Further, it is known that patients with cognitive impairment tend to underestimate their level of apathy due to anosognosia. Therefore, it is very important to have the assessment of eg, the caregiver.^40^, ^41^

Although a higher cognitive deficit, especially in the executive domain, was identified in patients with PSP‐RS in several studies.^35^, ^42^, ^43^ Horta‐Barba and colleagues,^44^ reported that no significant difference was observed in cognitive performance between the phenotypes. It is important to note that no study has compared all phenotypes with one another, and therefore no precise conclusion can be drawn regarding the exact cognitive difference. This is an area that future studies may wish to explore further.

We could not detect a significant change over time in all items. However, the ShoCo scale in total and aphasia in particular revealed a significant change over time, which are promising results for detection of progressive cognitive and neuropsychiatric impairments that need to be investigated further in future studies.

### Limitations

Particularly emphasized must be that, while the ShoCo scale shows promise, it has not been directly tested on patients yet. Especially the advantage of a comprehensive short version in order to not overwhelm the patient might not have been fully appreciated by using the data of different scales originating from patient cohorts. This short scale on the other hand should help the physician with a quick and easy assessment of the patient's most relevant aspects of the cognitive state.

Another limitation is that due to the short form with five items, not all cognitive symptoms might be included. Consequently, this may result in an incomplete assessment of cognitive and neuropsychiatric status. To address this limitation, detailed assessments should be performed. Nevertheless, the ShoCo scale will be an ideal assessment tool to test relevant cognitive and neuropsychiatric decline in a patient centered manner.

Due to the small number of vPSP patients, we did not subdivide the patients into subcortical and cortical phenotypes.^45^ It is therefore possible that further cognitive differences may exist between the phenotypes.

Patients with severe aphasia should be evaluated cautiously, as aphasia could have an influence on other items such as bradyphrenia and apathy and thus more strongly influence the result of the total ShoCo scale. This has to be investigated in more detail in future studies to find out further cognitive and neuropsychiatric differences between these phenotypic groups.

This study indicates that the ShoCo scale can be used as a bedside tool for short and relevant cognitive and neuropsychiatric assessment of PSP patients. However, this study does not show a disease specificity, because only PSP patients have been studied with this scale so far.

With its time efficient and easy applicable form is has great potential to be used in clinical routine practice and trials as an outcome parameter for relevant cognitive and neuropsychiatric decline. Although more in‐depth research and applying the scale in daily clinical practice might be needed, the findings of this study show promising results for the comprehensive cognitive and neuropsychiatric assessment of PSP patients.

## Author Roles

(1) Research Project: A. Conception, B. Organization, C. Execution; (2) Statistical Analysis: A. Design, B. Execution, C. Review and Critique; (3) Manuscript: A. Writing the First Draft, B. Review and Critique.

S.P. 1B, 1C, 2A, 2B, 3A.

M.K.: 1A, 1B, 2A, 2C, 3B.

S.G.: 1B, 2A, 2B, 3B.

I.A.P.: 1A, 1B, 3B.

G.R.: 1A, 1B, 3B.

I.J.: 1C, 3B.

F.W.: 1A, 2A, 2C, 3B.

L.Y.: 1C, 3B.

L.K.: 1C, 3B.

M.H.: 1C, 3B.

M.P.N.: 1C, 3B.

M.Z.: 1C, 3B.

K.H.: 1C, 3B.

J.K.: 1C, 3B.

P.S.: 1C, 3B.

J.W.: 1C, 3B.

D.B. 1C, 3B.

S.P.: 1C, 3B.

L.T.: 1C, 3B.

D.G.: 1C, 3B.

F.G.: 1C, 3B.

W.H.J.: 1C, 3B.

A.A.K.: 1C, 3B.

I.C.: 1C, 3B.

T.W.: 1C, 3B.

D.J.P.: 1C, 3B.

C.E.: 1C, 3B.

C.T.: 1C, 3B.

J.C.: 1C, 3B.

J.S.: 1C, 3B.

A.Spottke.: 1C, 3B.

P.K.: 1C, 3B.

A.Schnitzler.: 1C, 3B.

A.Schneider.: 1C, 3B.

M.B.: 1C, 3B.

B.F.: 1C, 3B.

I.Z.: 1C, 3B.

M.B.: 1C, 3B.

E.W.: 1C, 3B.

J.L.: 1C, 3B.

S.K.: 1C, 3B.

E.D.: 1C, 3B.

W.G.: 1C, 3B.

S.T.: 1C, 3B.

I.K.: 1C, 3B.

J.P.: 1C, 3B.

T.G.: 1C, 3B.

K.B.: 1C, 3B.

A.S.: 1C, 3B.

A.E.: 1C, 3B.

G.C.P.: 1C, 3B.

G.U.H.: 1A, 1B, 2A, 2C, 3A, 3B.

## Disclosures

**Ethical Compliance Statement:** Ethics committee: Hannover Medical School, Carl‐Neuberg‐Straße 1, 30,625 Hannover, Lower Saxony, Germany. Ethics vote number: 3558‐2017; 23.06.2017. All patients or legal caregivers gave their written informed consent. We confirm that we have read the Journal's position on issues involved in ethical publication and affirm that this work is consistent with those guidelines.

**Funding Sources and Conflicts of Interest:** There are no funding sources or conflicts of interest relevant for this study to declare. The study cohort was supported by the German Center for Neurodegenerative Diseases (DZNE) and the German Parkinson's Association (DPG) to GUH and GR.

**Financial Disclosures for the Previous 12 Months:** MK serves as a consultant for Abbvie and Stada; received honoraria for scientific presentations from Abbvie, Neurodiem, Ever and Licher. MK was funded by the German Parkinson's disease association, MHH plus foundation (Hannover, Germany) and Petermax Müller Foundation (Hannover, Germany). GR is a full‐time employee at Roche Pharmaceuticals since July 2021 and has consulted for UCB, all outside of the submitted work. JK has received honoraria or consultation fees from AbbVie, Bial, Biogen, Desitin, Esteve, STADA, and Zambon; in addition, he is Specialty Chief Editor for Frontiers in Neurology (section Applied Neuroimaging) and Associate Editor (Neurology) for Therapeutic Advances in Chronic Disease. FG serves as a consultant for Abbvie and Stada; received honoraria for scientific presentations from Abbvie, Bial, Stada, Merz. IC serves as a consultant for Abbvie, Stadapharm and Desitin; received honoraria for scientific presentations from Abbvie, Bial, Stadapharm, Zambon; received funding from Merz. DP received honoraria as a speaker at symposia sponsored by Bial, Boston Scientific Corp., Medtronic Inc., AbbVie Inc., Zambon, and Esteve Pharmaceuticals GmbH. He received payments as a consultant for Boston Scientific Corp, AbbVie Inc., Bial, and Bayer, and he was awarded a scientific scholarship from Boston Scientific Corp. for a project titled “Sensor‐based optimization of Deep Brain Stimulation settings in Parkinson's disease” (Computergestützte Parameteroptimierung der Tiefen Hirnstimulation bei Patient:innen mit idiopathischem Parkinson‐Syndrom, COMPARE‐DBS). Finally, DP had his travel expenses reimbursed by Esteve Pharmaceuticals GmbH and Boston Scientific Corp for attending conferences. AS was supported by the Deutsche Forschungsgemeinschaft, and received consulting fees and/or speaker honoraria from Abbott, Abbvie, Alexion, bsh medical communication, Kyowa Kirin, Novartis, and Zambon.

## Supporting information


**TABLE S1.** Correlation of the individual PSP‐ShoCo Scale items with similar constructs.

## Data Availability

The data that support the findings of this study are available from the corresponding author upon reasonable request.
