# Supplementary material for: A Short Cognitive and Neuropsychiatric Assessment Scale for Progressive Supranuclear Palsy
Source: Mov Disord Clin Pract. 2025 Jan 27;12(6):764–74. doi: 10.1002/mdc3.14348 (PMC12187960; doi:10.1002/mdc3.14348)
Supplement: Supplementary file 1 — TABLE S1. Correlation of the individual PSP‐ShoCo Scale items with similar constructs. [file MDC3-12-764-s001.docx]

**Supplementary Table 1**

*Correlation of the individual PSP-ShoCo Scale items with similar constructs*

| Item | Scores measuring similar constructs | Spearman r |
| --- | --- | --- |
| **Bradyphrenia** | PSPRS part II. mentation  (sum of items 8 to 11) | 0.69*** |
|  | PSP-QoL item 41 | 0.38** |
|  | MoCA total score | -0.39** |
| **Apathy** | PSPRS item 1 (withdrawal) | 0.15* |
|  | PSP-QoL item 44  SAS total score | 0.25** |
|  |  | 0.59** |
| **Aphasia** | MoCA item (speech) | -0.40** |
| **Dysexecution** | MoCA item (visuospatial/ executive) | -0.3** |
| **Disinhibition** | PSPRS item 11 **(**grasping/imitatative/utilizing behavior) | 0.38** |
